# Rhomboid glossitis caused by candida?

**DOI:** 10.11604/pamj.2016.23.8.8671

**Published:** 2016-01-21

**Authors:** Inssaf Ramli, Badredine Hassam

**Affiliations:** 1 Department of Dermatology, Avicenna Hospital, Faculty of Medecine and Pharmacy, University Mohamed V, Souissi, Rabat, Morocco

**Keywords:** Rhomboid glossitis, plaque, tongue, candida

## Image in medicine

Rhomboid glossitis (RG), first described by Brocq in 1914, is an uncommon benign abnormality of the tongue, most frequently affecting men. His etiology is unknown, although it has been proposed that it may be derived from chronic candidiasis, or that it may be of embryological, inflammatory, or even immunological origin. It appears as a rounded or rhomboid painless plaque with well-defined margin, intense reddish or pinkish in colour due to atrophy or depapillation, and firm to palpation. The diagnosis of rhomboid glossitis is largely based on clinical examination, though histopathological studies of biopsies may be required if clinical appearance does not allow other possible diagnoses to be ruled out. No treatment is required for asymptomatic cases. We report a case of a female patient aged 24 years, consulted for a painless plaque in dorsum of the tongue. Clinical examination indicated an erythematous plaque, located anterior to the lingual “V” in left paramedial position, elongated and slightly fissured, about 2 x 0.5 cm in size. Candida culture tested positive. The lesion was thus diagnosed as RG with presence of C. albicans. Treatment was with fluconazole for 2 weeks at 50 mg per day. A complete disappearance of the lesion was noted. After 12 months follow-up, no recurrence was reported.

**Figure 1:**
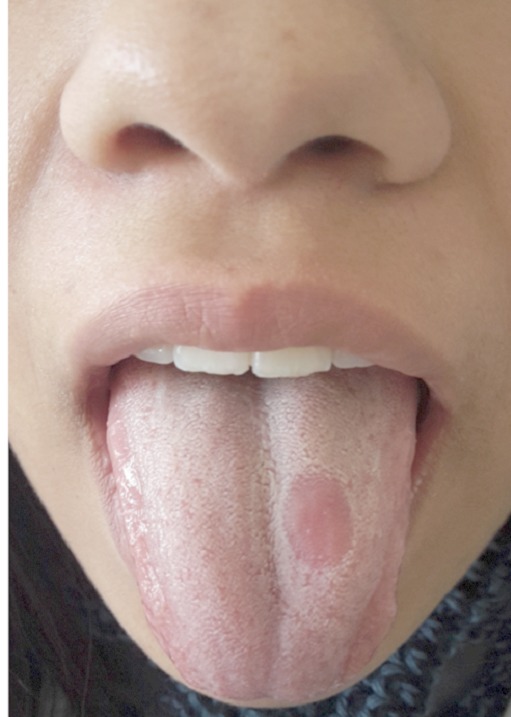
Paramedianrhomboid glossitis

